# *In Vitro* Activity of Pterocarpanquinone LQB-118 on *Trypanosoma cruzi* and Its Impact on Parasite Bioenergetics

**DOI:** 10.1021/acsomega.5c02858

**Published:** 2025-08-12

**Authors:** Bruno F. Azevedo, Andréia C. S. Brito, Johnata A. Vilarim, Bianca D. Ventura, Michely A. P. Mendes, Rosiane F. Dos Santos, Patrícia M. L. Dutra, Eduardo J. Lopes-Torres, Paulo R. R. Costa, Chaquip D Netto, Natália P. A. Nogueira, Silvia A. G. Da-Silva

**Affiliations:** † Laboratório de Imunofarmacologia Parasitária, 468988 Disciplina de Parasitologia/Faculdade de Ciências Médicas Universidade do Estado do Rio de Janeiro, Rio de Janeiro 20550-170, Brazil; ‡ Laboratório de Imunofisiologia do Exercício, Disciplina de Parasitologia/Faculdade de Ciências Médicas-Universidade do Estado do Rio de Janeiro, Rio de Janeiro 20550-170, Brazil; § Laboratorio de Helmintologia Romero Lascasas Porto Disciplina de Parasitologia/ Faculdade de Ciências Médicas, 28130 Universidade do Estado do Rio de Janeiro, Rio de Janeiro 20550-170, Brazil; ∥ Laboratório de Química Bioorgânica, Instituto de Pesquisas de Produtos Naturais, 28125 Universidade Federal do Rio de Janeiro, Rio de Janeiro 21941-941, Brazil; ⊥ Laboratório de Química, Universidade Federal do Rio de Janeiro, Campus Macaé, Rio de Janeiro 27930-560, Brazil; # Laboratório de Interação de Tripanosomatídeos e Vetores, Departamento de Bioquímica/Instituto de Biologia Roberto Alcantara Gomes-Universidade do Estado do Rio de Janeiro, Rio de Janeiro 20550-170, Brazil; ∇ Instituto Nacional de Ciência e Tecnologia-Entomologia Molecular-INCT-EM, Rio de Janeiro 21941-590, Brazil

## Abstract

The hemoflagellate protozoan *Trypanosoma cruzi* is the etiologic agent of Chagas disease, one of the neglected tropical diseases endemic to Latin America with a high socioeconomic impact. Treatment remains restricted to two drugs, benznidazole and nifurtimox, which present several side effects and are ineffective in the chronic phase of the disease. The synthetic pterocarpanquinone LQB-118, a hybrid molecule synthesized from lapachol and pterocarpan, exhibits several biological activities, including antiparasitic effects similar to those of its precursors. The present study aimed to investigate the *in vitro* activity of LQB-118 on *T. cruzi* and its effect on the parasite’s mitochondrion. For an initial evaluation of the antiparasitic effect, intracellular amastigotes, epimastigotes, and metacyclic trypomastigotes were incubated with LQB-118, and the IC_50_ values were 4.2, 2.5, and 38.1 μM, respectively. DNA fragmentation analysis by TUNEL labeling showed that treatment with LQB-118 induced selective fragmentation of amastigote nuclei, while macrophage nuclei remained intact. In addition, treatment of metacyclic trypomastigotes with LQB-118 reduced the infectivity of peritoneal macrophages. LQB-118 also induced changes in parasite mitochondrial function, causing H_2_O_2_ production and increasing O_2_ consumption in mitochondrial respiration states. Corroborating these results, LQB-118 exposure led to severe ultrastructural disruption, especially in the parasite’s single mitochondrion, which exhibited swelling and disorganization. Additionally, other cellular changes, such as autophagic vacuoles, nuclear chromatin condensation, and shrinkage, were observed. These results indicate that LQB-118 exerts its antiparasitic action on *T. cruzi* by disrupting mitochondrial physiology, leading to mitochondrial reactive oxygen species production, oxidative stress, and parasite death.

## Graphical abstract

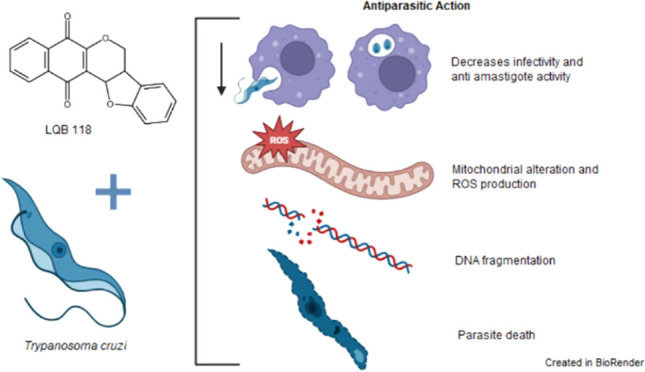

## Introduction

Chagas disease, or American trypanosomiasis, is a neglected disease caused by the protozoan *Trypanosoma cruzi*, endemic in 21 Latin American countries, mainly in poor rural areas. Still, due to migratory flows, the disease is diagnosed in other regions of the world.^1,2^ This illness affects about 6–7 million people and causes approximately 12,000 deaths every year.^3^ *T. cruzi* belongs to the order Kinetoplastea and the family Trypanosomatidae, displaying three distinct biologically relevant forms: epimastigotes, which multiply by binary division inside the invertebrate host; trypomastigotes, nondividing infective forms found in both the invertebrate vector and mammalian host; and amastigotes, which are intracellular in mammalian host cells and multiply by binary division.^4^

*T. cruzi* is classically transmitted during a triatomine vector blood meal. However, entomological surveillance programs have brought this transmission route under control.^5^ On the other hand, the incidence of infection via the oral route has increased in recent years due to the consumption of contaminated food such as açaí and sugar cane juice.^6^ Other transmission routes include blood transfusion, organ transplantation, and vertical transmission.^7^

The clinical course of Chagas disease comprises acute and chronic phases. The acute phase is usually mildly symptomatic and characterized by high parasitemia. In the indeterminate, asymptomatic phase, the infected individual presents low parasitemia and remains seropositive for years or decades. In the chronic symptomatic phase, 20–30% of patients may develop cardiac symptoms (including left ventricular systolic dysfunction, dilated cardiomyopathy, arrhythmias, thromboembolic events, and terminal heart failure) or digestive symptoms (such as megaesophagus and megacolon).^2,8^

Benznidazole and nifurtimox are the current drugs used to treat Chagas disease. However, due to long treatment regimens, numerous side effects, and low tissue penetration, their efficacy against intracellular amastigotes is limited, making treatment of the chronic phase unsatisfactory.^9,10^ Therefore, it remains imperative to develop new drugs that are less toxic and effective in the chronic phase of Chagas disease.

In this context, several data are available on the characterization of natural products, as naphthoquinones and pterocarpans, which have cytotoxic activity against *T. cruzi*, impairing epimastigote proliferation and metacyclic trypomastigote viability.^11–13^ The pterocarpanquinone LQB-118 was synthesized by the hybridization of lapachol (a naphthoquinone) and a pterocarpan (an isoflavonoid). LQB-118 has been widely studied over the years and has shown antitumoral activity, inducing oxidative stress and apoptosis in leukemic cells,^14,15^ and has also exhibited antiparasitic activity against *Toxoplasma gondii* ^16^ and different *Leishmania* species.^17–19^ The antileishmanial activity of LQB-118 suggested the parasite’s single mitochondrion as an important target, inducing intracellular ROS production, depolarization of the mitochondrial membrane potential (ΔΨ_m_), mitochondrial swelling, and phosphatidylserine externalization, leading to apoptosis.^17,18^

Mitochondria are organelles that play a pivotal role in cellular respiration, regulating the cell cycle, proliferation, apoptosis, oxidative phosphorylation, and ATP production coupled with the electron transport system.^20,21^ Unlike other eukaryotic cells, trypanosomatids such as *Leishmania* sp. and *T. cruzi* have a single mitochondrion, and cell survival depends on the functionality of this organelle, making it an attractive target for the research of molecules with antiparasitic activity.^22,23^

Since the antileishmanial mode of action of LQB-118 targets the parasite’s mitochondrion, its antiparasitic activity may be extended to other trypanosomatids such as *T. cruzi*. Thus, in the present study, we demonstrate the *in vitro* activity of LQB-118 against the three most relevant forms of *T. cruzi*, decreasing the infectivity of metacyclic trypomastigotes, the infection index, and inducing DNA fragmentation in intracellular amastigotes. LQB-118 also induced severe morphological and functional alterations in the *T. cruzi* mitochondrion, leading to ultrastructural changes, decreased oxygen consumption, and oxidative stress

## Results

### Susceptibility of *T. cruzi* to LQB-118

Before investigating the antiamastigote activity, the toxicity of LQB-118 (0–80 μM) to peritoneal macrophages was evaluated after 72 h of treatment. LQB-118 induced a loss of viability only at higher concentrations, starting at 40 μM compared to DMSO, significantly reducing viability by 78% at the highest concentration tested, 80 μM (Figure 1A).

**Figure 1:**
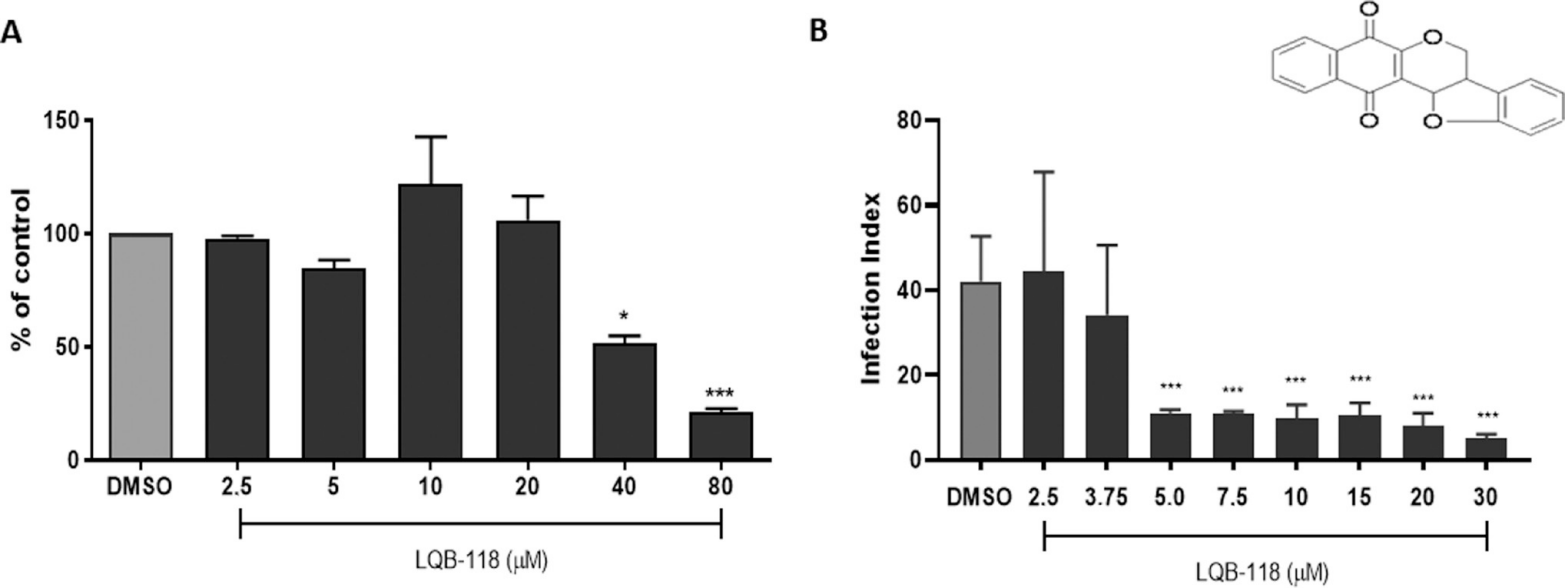
*In vitro* activity of LQB-118 on intracellular amastigote of *T. cruzi* and macrophage toxicity. (A) Uninfected peritoneal macrophage monolayers were treated with the indicated concentrations of LQB-118 for 72 h/37 °C and cell viability was determined by MTT. (B) Peritoneal macrophage monolayers were infected with trypomastigotes (ratio 5:1) and treated with the indicated concentrations of LQB-118 for 72 h/37 °C. After staining, the Infection Index was determined by counting at least 100 macrophages. Controls were macrophage monolayers infected or not incubated with RPMI plus 0.2% DMSO. The data presented are representative of three independent experiments performed in triplicate. Mean ± SD, *n* = 3. *p < 0.05, ***p < 0.001 (in relation to Control).

The effect of LQB-118 on intracellular amastigotes was evaluated by incubating monolayers of infected macrophages with LQB-118 (0–30 μM) for 72 h. LQB-118 significantly decreased the infection index starting at 5 μM (73% reduction), reaching an 88% reduction at 30 μM, compared to the control (Figure 1B).

Epimastigotes were treated with various concentrations of LQB-118 (0–30 μM) for 96 h, and the number of parasites was counted daily. We observed a decrease in epimastigote growth relative to the control starting at 72 h and established 96 h as the time point for IC_50_ determination. Metacyclic trypomastigotes were treated with LQB-118 at different concentrations (0–30 μM) for 48 h, after which motile parasites were counted. The IC_50_ values for the different developmental forms of *T. cruzi* and the CC_50_ for peritoneal macrophages are shown in Table 1. The IC_50_ for the replicating forms—epimastigotes (2.5 μM) and amastigotes (4.2 μM)—were lower than for the nonreplicating trypomastigote form (40 μM).

**Table 1:** LQB-118 Inhibitory Activity for *T. cruzi* and Toxicity to Macrophages^a^

molecule	epimastigotes IC_50_/96 h (μM)	metacyclic trypomastigotes IC_50_/48 h (μM)	intracellular amastigotes IC_50_/72 h (μM)	peritoneal macrophages CC_50_/72 h (μM)
LQB-118	2.5 ± 0.3	40 ± 2.6	4.2 ± 0.7	40 ± 0.5
benznidazole	6.1 ± 0.7	22.75 ± 3.1	21.21 ± 0.1	396 ± 5.7

a IC_50_ half-maximal inhibitory concentration; CC50 half-maximal cytotoxic concentration. Data represents the mean of three independent experiments performed in triplicate. Mean ± SD, *n* = 3.

When comparing the activity of LQB-118 with the reference drug benznidazole, we observed that the IC_50_ of LQB-118 in amastigotes was lower (4.2 μM) than that of benznidazole (21.2 μM). The same was observed in epimastigotes. However, in trypomastigote forms, benznidazole was more active (Table 1).

### Treatment with LQB-118 Induces DNA Fragmentation in Intracellular Amastigotes

To evaluate whether the antiamastigote effect of LQB-118 could induce DNA fragmentation in intracellular amastigotes, infected peritoneal macrophages were treated with LQB-118 for 72 h. A TUNEL assay and fluorescence microscopy were used to detect DNA fragmentation.

Figure 2 shows that 4.2 μM LQB-118 (the IC_50_ for intracellular amastigotes) and 15 μM (approximately three times the IC_50_) were able to induce selective DNA fragmentation in the parasite without affecting macrophage DNA. Positive controls treated with DNase showed DNA fragmentation in both the parasite and the macrophage (Figure 2).

**Figure 2:**
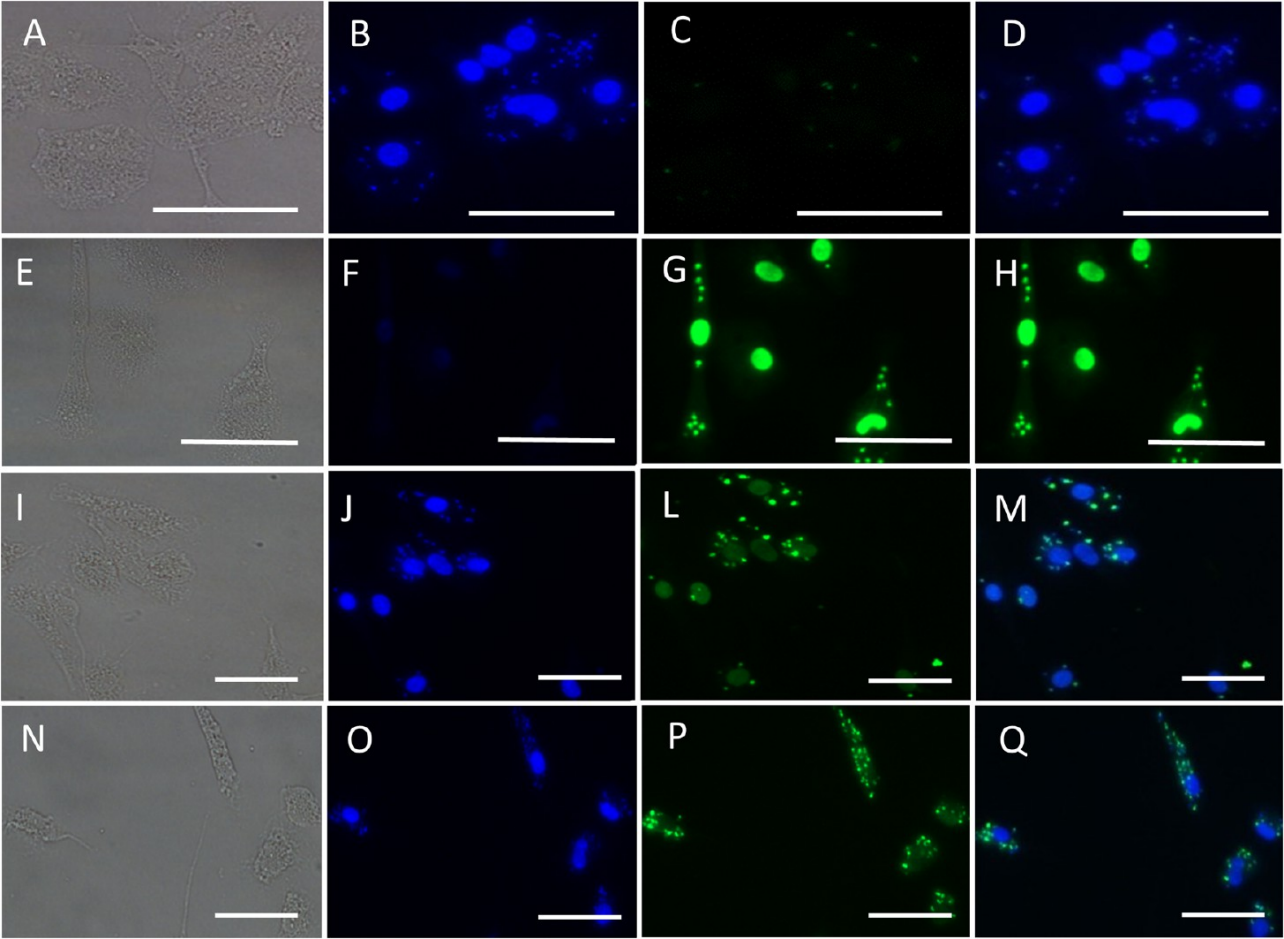
Intracellular amastigote DNA fragmentation induced by LQB-118. Infected peritoneal macrophage monolayers were treated with the indicated concentrations of LQB-118 for 72 h/37 °C. Monolayers were labeled using TUNEL and observed by fluorescence microscopy. Controls were macrophage-infected incubated with RPMI plus 0.2% DMSO or DNase. DIC (A, E, I, N), DAPI (B, F, J, O), TUNEL (C, G, L, P), DAPI+TUNEL (D, H, M, Q). Control with DMSO (A–D), Control with DNase (E–H), LQB-118 4.2 μM (I–M), LQB-118 15 μM (N–Q). Images were presented in 400× magnification and representative of three independent experiments performed in triplicate. *n* = 3.

### LQB-118 Decreases the Infectivity of Metacyclic Trypomastigotes in Mammalian Cells

Since LQB-118 had a relatively low effect on the viability of trypomastigote forms, we investigated whether it would impact their ability to invade host cells. To evaluate whether LQB-118 treatment could affect trypomastigote infectivity, parasites were preincubated with LQB-118 for 48 h, and then peritoneal macrophage monolayers were infected with the treated parasites.

Images show that after 24 h of interaction, some pretreated parasites were visualized outside the macrophages, apparently unable to infect host cells at 10 and 40 μM LQB-118 (Figure 3A). In the control group using untreated trypomastigotes, few parasites were observed outside the macrophages. After 72 h of infection—enough time for differentiation into amastigotes and their multiplication—fewer intracellular amastigotes were observed compared to the control (Figure 3B). In Figure 3C,D, we quantified the number of intracellular amastigotes and observed a significant reduction in the infection index at both 24 h (Figure 3C) and 72 h (Figure 3D) after infection. These results indicate that LQB-118 decreases the capacity of trypomastigotes to infect peritoneal macrophages.

**Figure 3:**
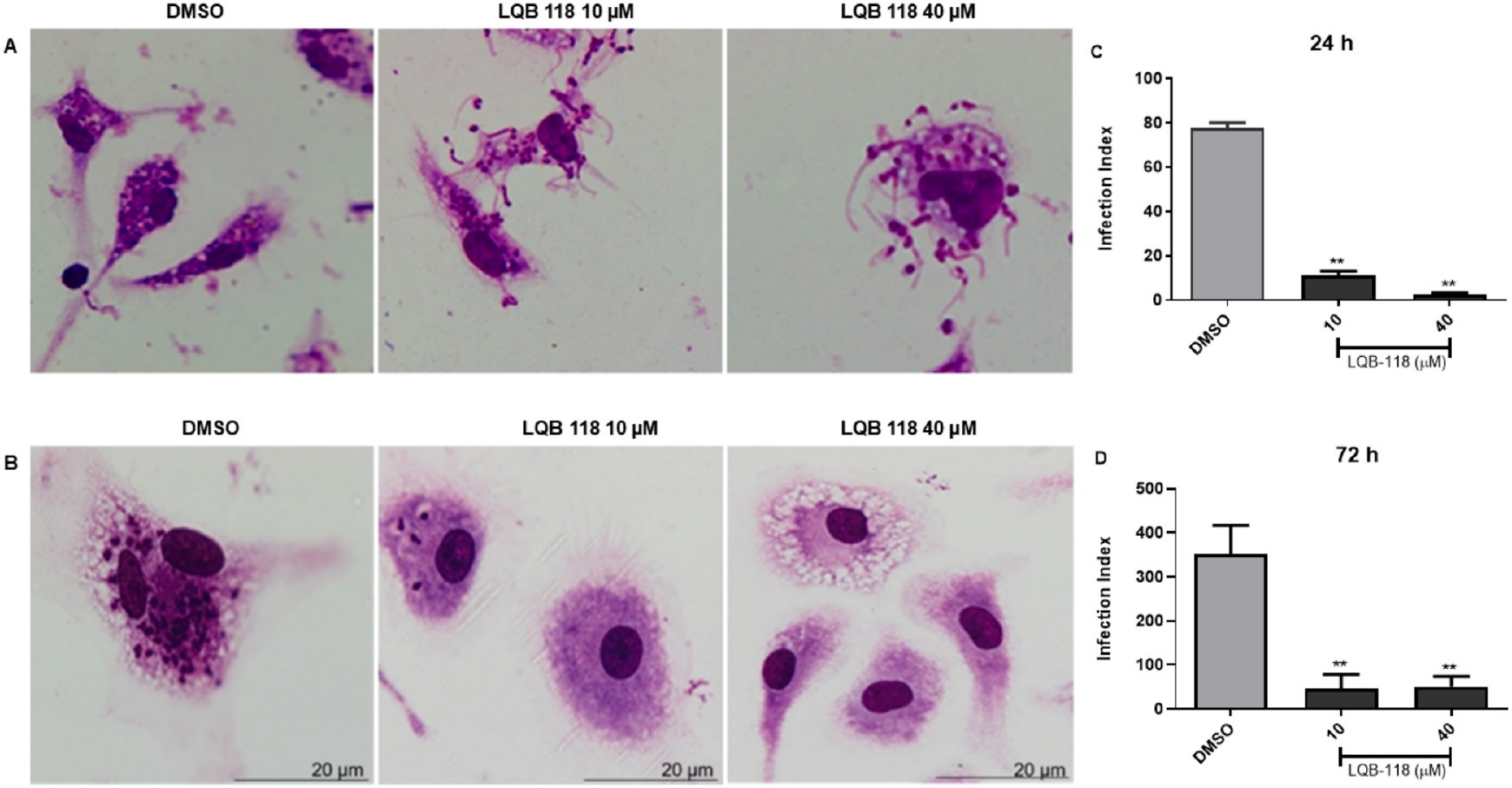
Effect of LQB-118 on the infectivity of metacyclic trypomastigotes. Metacyclic trypomastigotes pretreated with LQB-118 (10 or 40 μM) or 0.2% DMSO for 48 h/27 °C were used to infect peritoneal macrophages monolayers (ratio 5:1). Cells were stained and amastigotes were counting at least 100 macrophages at 24 or 72 h. Images were obtained after 24 h (A) and 72 h (B) of infection. Infection Index evaluated after 24 h (C) and 72 h (D). Data representative of three independent experiments performed in triplicate. Mean ± SD, *n* = 3. **p < 0.05 (difference compared to DMSO). Bars = 20 μm.

### LQB-118 Alters O_2_ Consumption in Treated Epimastigotes

Epimastigotes were treated with LQB-118 for 24 and 48 h, followed by high-resolution respirometry analysis of mitochondrial respiration. Treatment for 24 h with 2.5 μM LQB-118 significantly increased oxygen consumption in different respiratory states (Figure 4A–L).

**Figure 4:**
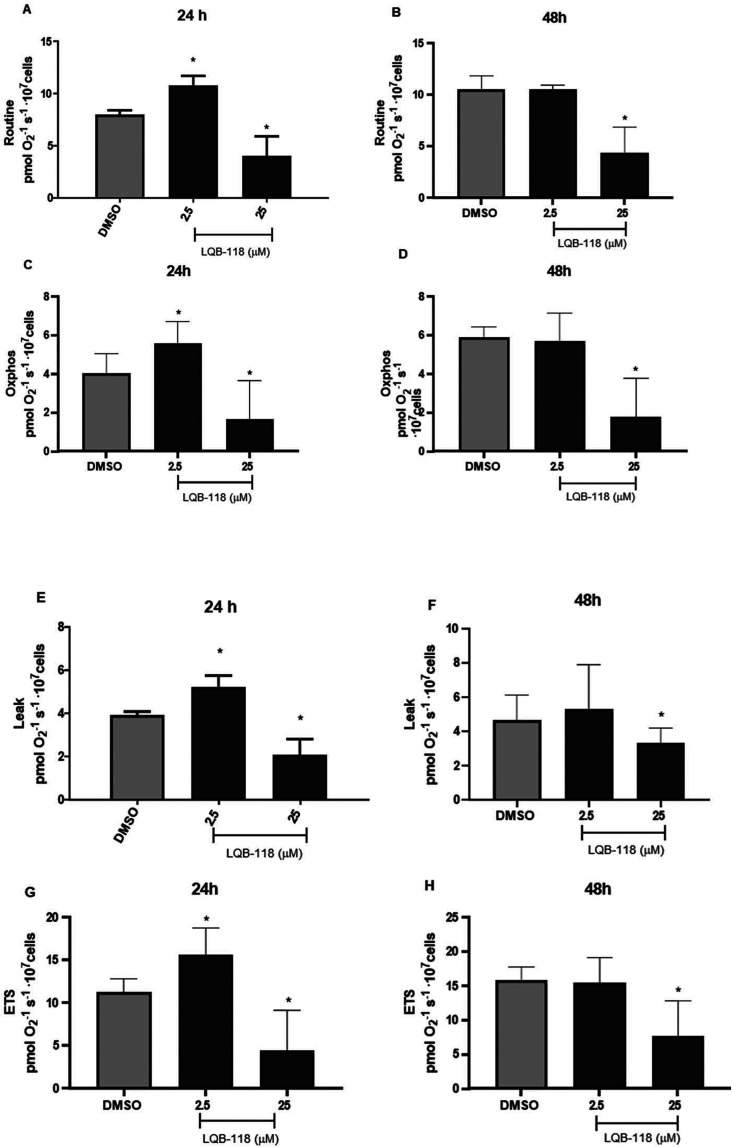

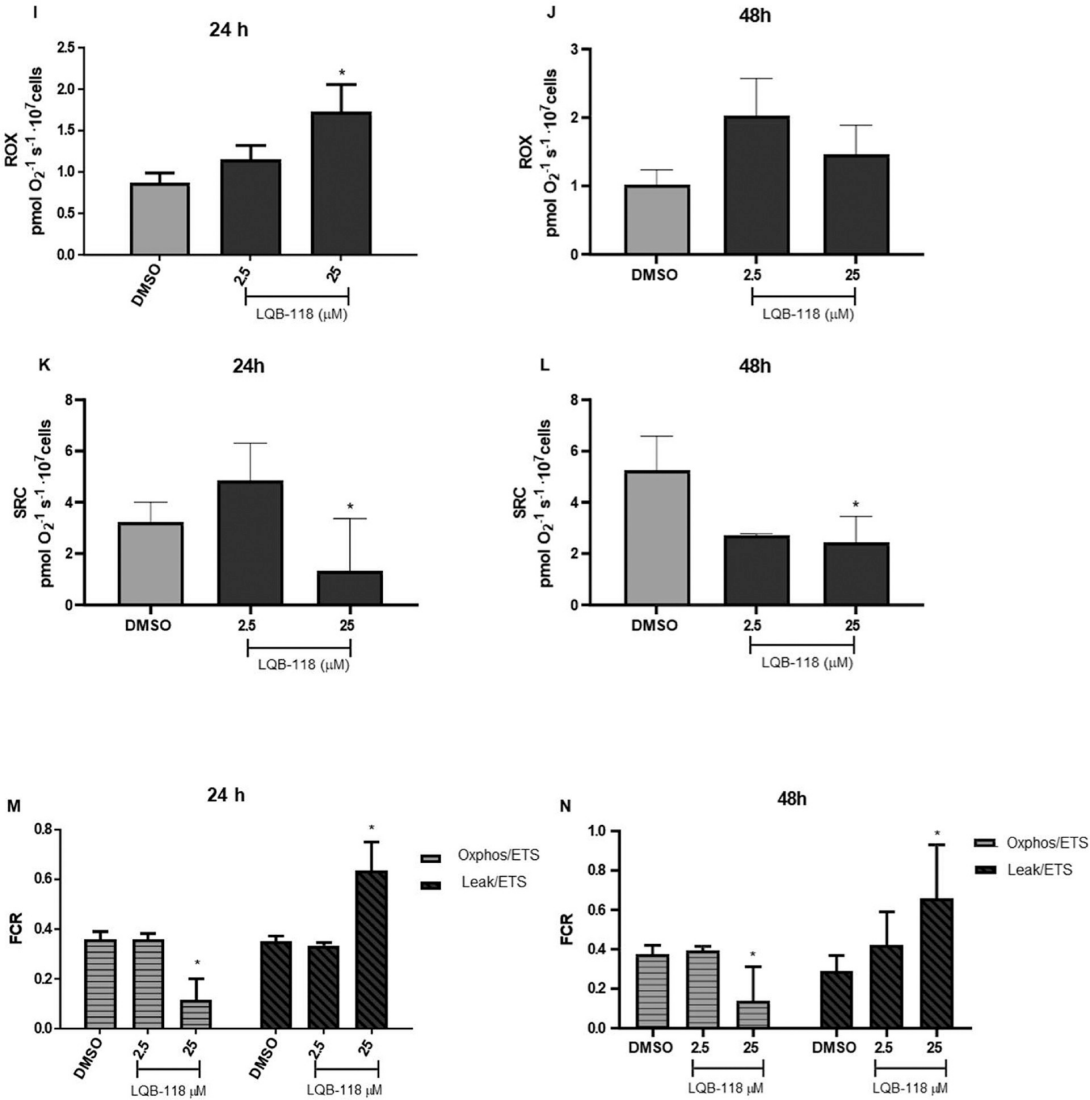
Impact of LQB-118 on oxygen consumption. Epimastigotes were treated in the presence or absence of LQB-118 for 24–48 h/27 °C and then submitted to high-resolution respirometry. Control was parasites incubated in BHI medium with 0.2% DMSO. (A) Routine (physiological respiration) 24 h. (B) Routine 48 h. (C) Oxphos 24 h. (D) Oxphos 48 h. (E) Leak 24 h. (F) Leak 48 h. (G) ETS 24 h. (H) ETS 48 h. (I) ROX 24 h. (J) ROX 48 h. (K) SCR 24 h. (L) SCR 48 h. (M) FCR 24 h. (N) FCR 48 h. Data representative of four independent experiments performed in triplicate. Mean ± SD, *n* = 4. *p < 0.05 (difference compared to DMSO).

Analyzing oxygen consumption linked to OXPHOS, the capacity for ATP synthesis via oxidative phosphorylation increased by 30% compared to the control. This effect was restored to normal levels after 48 h of treatment, as was physiological respiration (routine).

Subsequently, we compared proton leak (after the addition of oligomycin) and the maximum electron transfer capacity (ETS) (after the addition of FCCP) between treated and untreated parasites after 24 h, and observed significant increases of 25 and 29%, respectively. The flow of electrons in the mitochondrial electron transport system increased, probably due to changes in substrate metabolism.^27,28^

Forty-eight h of parasite treatment showed no significant differences. ROX and SCR also did not significantly differ from those in untreated parasites treated with 2.5 μM LQB-118 at either 24 or 48 h.

When parasites were incubated with 25 μM LQB-118, oxygen consumption in different respiratory states significantly decreased at both time points (Figure 4A–L). There was a reduction in ATP synthesis capacity via OXPHOS by 42 and 70% at 24 and 48 h, respectively, compared to untreated parasites. Proton leak decreased by 47 and 28%.

Regarding ETS, the decrease was 61 and 51%, suggesting a loss of integrity in the electron transport system.^28^ Additionally, there was a significant increase of 56% in nonmitochondrial oxygen consumption (ROX) at 24 h. The spare respiratory capacity (SRC) also decreased significantly—by 58 and 53% at 24 and 48 h, respectively—indicating that the extra mitochondrial capacity to produce energy was compromised.

Although treatment with 2.5 μM LQB-118 did not alter the flux control ratio (FCR) compared to untreated parasites, treatment with 25 μM significantly changed the FCR at both time points, decreasing FCR (OXPHOS/ETS) and increasing FCR (Leak/ETS) (Figure 4M–N).

These results demonstrate that LQB-118 modulates mitochondrial oxygen consumption in epimastigotes: initially increasing consumption at 2.5 μM and decreasing it at 25 μM.

### Treatment with LQB-118 Induces H_2_O_2_ Production

To evaluate whether treatment with LQB-118 leads to H_2_O_2_ production, epimastigotes were treated with LQB-118 for 24 and 48 h. Then, the analysis was performed using the Amplex Red probe. The results show increased H_2_O_2_ production at both time points and LQB-118 concentrations compared to the DMSO control (Figure 5A,B).

**Figure 5:**
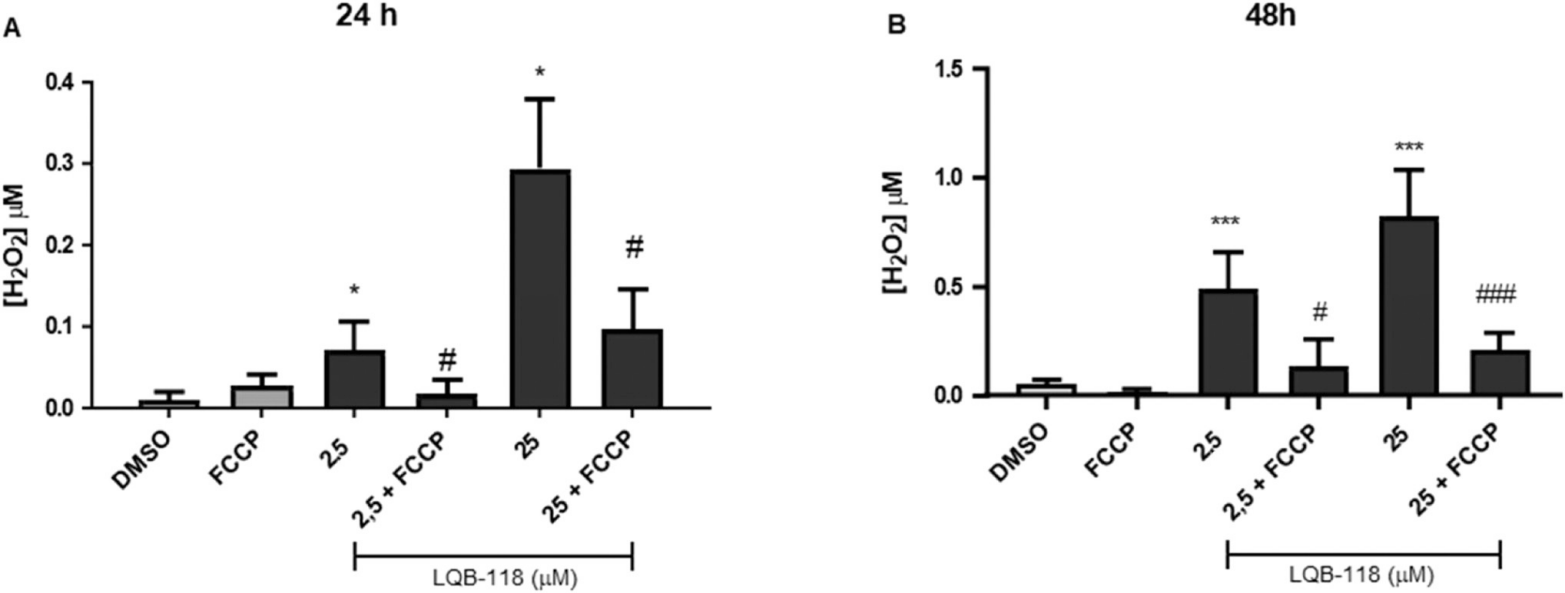
Mitochondrial production of H_**2**_O_2_ in parasites treated with LQB-118. Epimastigotes were treated in the presence or absence of LQB-118 for 24 and 48 h/27 °C. Controls were parasites incubated in BHI medium plus 0.2% DMSO or mitochondrial uncoupler FCCP. Hydrogen peroxide was measured by the Amplex Red probe and the results expressed an increase in H_2_O_2_ production relative to control (DMSO) or a decrease between LQB-118 + FCCP and LQB-118 alone. (A) Treatment with LQB-118 for 24 h. (B) Treatment with LQB-118 for 48 h. Data representative of three independent experiments performed in triplicate. Mean ± SD, *n* = 3. *p < 0.05, ***p < 0.001 (difference from DMSO). #*p* < 0.05, ### *p* < 0.001 (difference compared to LQB-118).

To assess whether the H_2_O_2_ produced was mitochondrial, the mitochondrial uncoupler FCCP was added to stimulate maximum respiration uncoupled from ATP synthesis, thereby decreasing ROS production.^29,30^ When FCCP was added together with LQB-118, the H_2_O_2_ production stimulated by LQB-118 decreased by 72 and 70%, compared to 2.5 and 25 μM LQB-118 at 24 h, and by 74 and 75% at 48 h, respectively, indicating that a large proportion of the H_2_O_2_ produced was indeed mitochondrial.

### LQB-118 Causes Ultrastructural Changes in Treated Epimastigotes

To evaluate whether treatment with LQB-118 could induce changes in the external and intracellular ultrastructure of epimastigotes, the parasites were treated with LQB-118 for 48 h, and scanning (SEM) and transmission (TEM) electron microscopy were performed.

In the analysis of cell topography, the control group showed the typical elongated cell body with flagellum and intact plasma membrane, without morphological changes, as expected (Figure 6A–C). In contrast, parasites treated with 2.5 μM LQB-118 displayed a shrinkage pattern (Figure 6B), flagellum detachment from the cell body (Figure 6D), and cell rounding (Figure 6E), which was more pronounced in parasites treated with 25 μM LQB-118 (Figure 6F).

**Figure 6:**
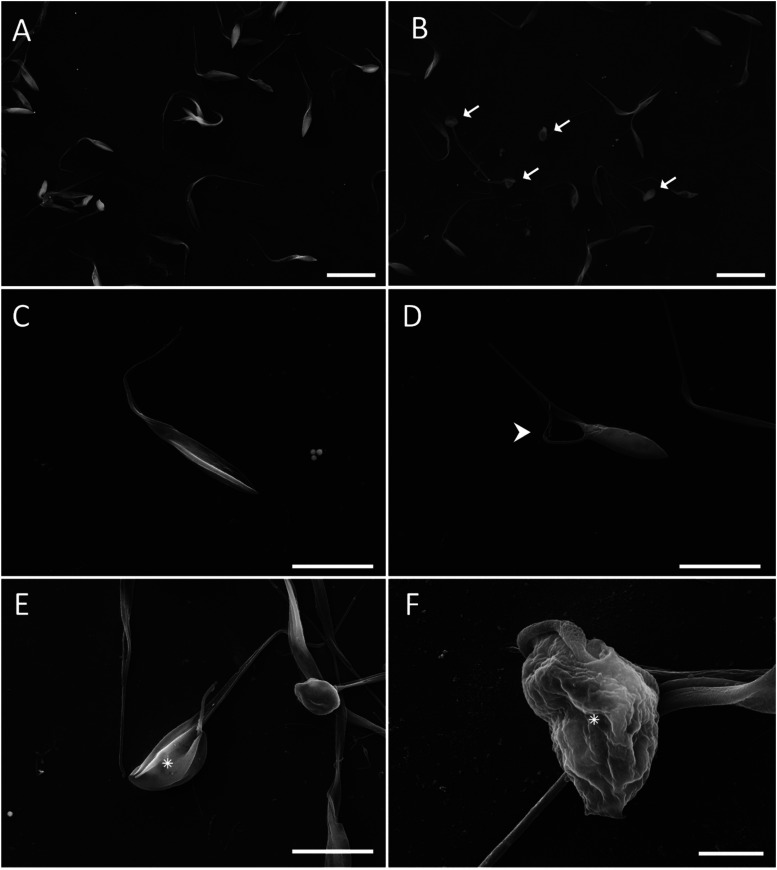
Ultrastructural change caused by LQB-118 on *T. cruzi* based on scanning electron microscopy. Epimastigote were treated with LQB-118 for 48 h/27 °C and analyzed on scanning electron microscopy. (A, C) show control, in which epimastigotes were incubated in BHI medium containing 0.2% DMSO, presenting the typical morphology of the parasites. (B, D, E) Epimastigotes treated with 2.5 μM LQB-118. (F) Epimastigotes treated with 25 μM LQB-118. In (B) arrow indicates cell shrinkage. Detachment of flagellum is seen (D, arrowhead). Asterisk show rounding up of cells (E and F). Bars = 10 μm (A, B); 5 μm (C–E); 1 μm (F). Results are representative of two independent experiments.

Internal ultrastructural characterization showed that control parasites exhibited typical epimastigote structures, with preserved nucleus, mitochondria, and kinetoplast, as expected (Figure 7A–B). In contrast, parasites treated with 2.5 μM LQB-118 showed nuclear chromatin condensation, disorganized Golgi complex (Figure 7C), swollen mitochondria (Figure 7E), and the presence of autophagic-like vacuoles (Figure 7D), which were also observed in parasites treated with 25 μM LQB-118 (Figure 7F). These changes suggest that the toxicity of LQB-118 to the parasite may involve both apoptotic and autophagic cell death.

**Figure 7:**
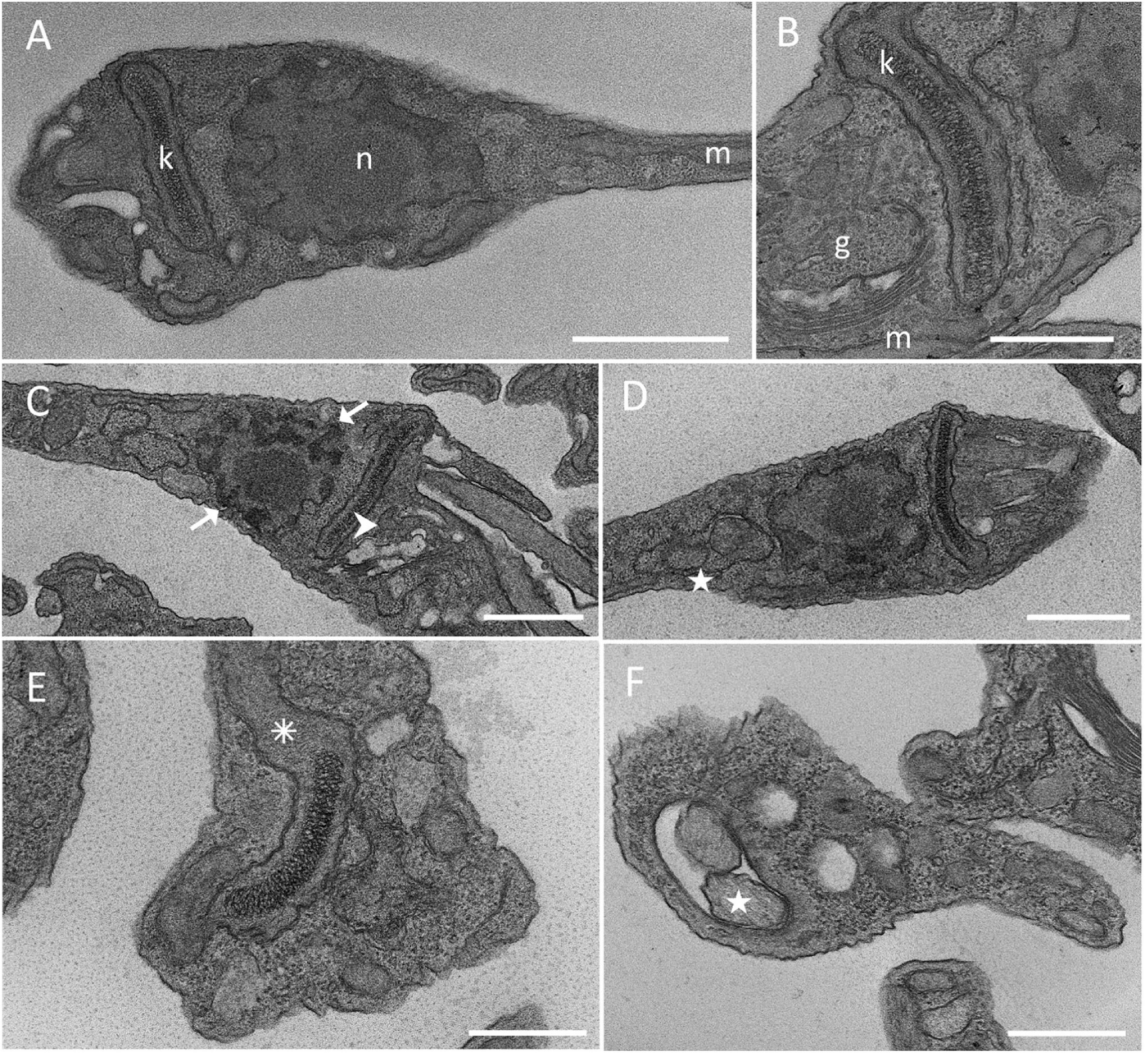
Ultrastructural analysis of changes induced by LQB-118 based on transmission electron microscoscopy. Epimastigotes were incubated with LQB-118 for 48 h/27 °C and analyzed by transmission electron microscopy. (A, B) Control incubated with BHI medium containing 0.2% DMSO, showing the typical aspect of the structures of parasite. (C–E) Epimastigotes treated with 2.5 μM LQB-118. (F) Epimastigotes treated with 25 μM LQB-118. Arrows indicate condensed chromatin nuclei and Golgi complex disarranged, observed in (C) (arrowhead). LQB-118 treatment also induces the formation of autophagic vacuole profiles (D, F - star) and swollen mitochondria (E, asterisk). Abbreviation: n - nucleus; m - mitochondria; k - kinetoplast; g - Golgi complex. Bars = 1 μm (A, C, D); 500 nm (B, E, F). Results are representative of two independent experiments.

## Discussion

The treatment of Chagas disease remains a challenge, especially in the chronic phase, which lacks a drug with satisfactory activity.^9,10^ Therefore, research focused on discovering new drugs with improved efficacy is necessary.

In the present study, we demonstrated for the first time the *in vitro* activity of the pterocarpanquinone LQB-118 against *T. cruzi*. The antiparasitic activity of LQB-118 was observed in intracellular amastigotes (IC_50_ = 4.2 ± 0.7 μM), with relatively low toxicity to mammalian cells (CC_50_ = 40 ± 0.5 μM). We showed that treatment of infected macrophages with LQB-118 selectively induced DNA fragmentation in intracellular amastigotes, without affecting the host cell. Since amastigotes are the most clinically relevant targets for anti-*T. cruzi* drugs, including in the chronic phase of Chagas disease, these findings suggest a promising potential for LQB-118. Our group previously demonstrated the antiamastigote activity of LQB-118 against the trypanosomatid *Leishmania braziliensis* (IC_50_ = 7.5 ± 0.8 μM), which was also associated with DNA fragmentation in amastigotes.^18^

Interestingly, we did not observe a pronounced activity of LQB-118 on the viability (motility) of trypomastigotes (IC_50_ = 40 ± 2.6 μM). However, LQB-118 treatment significantly reduced the infectivity of these trypomastigotes for macrophages Coumarins, a natural product, have also been shown to decrease the infectivity of pretreated trypomastigotes to Vero cells, associated with a reduction in *T. cruzi* surface protein expression responsible for host cell adhesion, such as mucin-like proteins (TcMUC II), mucin-associated surface proteins (MASPs), and trypomastigote small surface antigen (TSSA).^31^ In our light microscopy analysis, we observed several pretreated trypomastigotes adhered to the surface of peritoneal macrophages but not invading the cells. Although further studies are needed, LQB-118 may alter the surface molecules of trypomastigotes, contributing to the reduction of their ability to infect mammalian cells.

After demonstrating the antiparasitic activity of LQB-118 against *T. cruzi*, we set out to delineate its mode of action. The DNA fragmentation observed in intracellular amastigotes treated with LQB-118 is a hallmark of apoptotic cell death.^32^

In epimastigotes treated with LQB-118 and analyzed by SEM and TEM, we observed other typical features of apoptosis, such as cell shrinkage, cell rounding, and chromatin condensation. Similar effects were observed in *Leishmania amazonensis*, in which LQB-118 caused DNA fragmentation in intracellular amastigotes and, in promastigotes, induced a rounded morphology, chromatin condensation, and mitochondrial membrane potential (ΔΨ_m_) depolarization, leading to apoptosis-induced cell death.^17^

We also observed the presence of structures resembling autophagic vacuoles in epimastigotes. It has been demonstrated that, in epimastigotes treated with a β-lapachol derivative, features of both apoptotic and autophagic cell death were induced, such as DNA fragmentation, phosphatidylserine externalization, and autophagosome formation.^13^ Therefore, additional studies are needed to elucidate whether the toxicity of LQB-118 to *T. cruzi*, in addition to inducing apoptosis, may also involve autophagy.

As observed in *Leishmania* sp.,^17,18^ our data indicated significant changes in the ultrastructure of *T. cruzi* mitochondria, suggesting that this may be the target organelle of LQB-118 in trypanosomatids. LQB-118 increased ROS production of mitochondrial origin, as observed when associated with FCCP, an uncoupler of oxidative phosphorylation. FCCP dissipates the proton gradient across the inner mitochondrial membrane, increasing mitochondrial oxygen consumption, which leads to less electron leakage and, consequently, decreased ROS production.^33^ The increased H_2_O_2_ production was accompanied by a significant initial increase in oxygen consumption at different respiratory states with 2.5 μM LQB-118 (IC_50_). Another study showed that treatment of epimastigotes with H_2_O_2_ caused nuclear and mitochondrial DNA damage, where repair was accompanied by increased oxygen consumption to maintain ATP levels.^34^ It is plausible to suggest that H_2_O_2_ production in LQB-118-treated epimastigotes may have caused nuclear and mitochondrial DNA damage; consequently, compensatory mechanisms were triggered to maintain ATP levels to assist in DNA repair. However, further studies involving nuclear and mitochondrial DNA damage in LQB-118-treated epimastigotes are necessary. When treated with 25 μM LQB-118 (10-fold the IC_50_), we observed a significant decrease in oxygen consumption, which may result in intense ROS production and the pathological phenomenon of oxidative stress, which, in turn, can lead to the loss of protein and lipid function and DNA lesions.^35^ A study conducted with naphthoquinone derivatives in *T. cruzi* epimastigotes demonstrated that these molecules were able to induce mitochondrial dysfunction by inhibiting the mitochondrial electron transport chain, specifically at the level of complex II–III, causing a collapse of the mitochondrial membrane potential and reduced oxygen consumption, leading to increased ROS production.^36^

This suggests that oxidative stress indeed occurred in epimastigotes treated with 25 μM LQB-118, resulting in the loss of protein function, inhibition of the mitochondrial electron transport chain with a consequent reduction in oxygen consumption, and a decrease in ATP synthesis capacity via OxPHOS. Furthermore, due to the low spare respiratory capacity (SRC), the parasites lacked the additional mitochondrial capacity to produce energy and cope with oxidative stress.

Regarding the modulation of parasite bioenergetics by LQB-118, we also observed a low FCR (OxPHOS/ETS) and a high FCR (Leak/ETS), indicating that the mitochondrial electron transport chain and oxygen consumption were correlated with proton leak, with a decoupling between oxidative phosphorylation and the electron transport system, ultimately affecting ATP synthesis.

Due to their chemical structure, naphthoquinones can interfere with mitochondrial electron flow by diverting electrons from the total pool of ubiquinones. Menna-Barreto and colleagues proposed that naphthoquinones are reduced in the *T. cruzi* mitochondrion to the semiquinone radical, which is not oxidized and accumulates in the inner mitochondrial membrane, where it reacts directly with molecular oxygen to produce superoxide anion. This superoxide can then be converted to H_2_O_2_ by superoxide dismutases.^36^

In this way, the high production of reactive oxygen species leads to oxidative stress, causing deleterious effects on the parasite. Since the pterocarpanquinone LQB-118 is a naphthoquinone, a similar process may have occurred. Following this reasoning, LQB-118 would be reduced to the semiquinone radical in the inner mitochondrial membrane, reacting with oxygen and leading to ROS production and oxidative stress. This, in turn, could result in damage to mitochondrial complexes, leading to depolarization of ΔΨ_m_, changes in oxygen consumption, and decreased ATP synthesis—a phenomenon previously observed in *Leishmania* sp.^17,18^

However, further studies are needed to evaluate damage to mitochondrial complexes and ΔΨ_m_ depolarization following LQB-118 treatment. This oxidative stress would also lead to extra-mitochondrial effects, such as DNA fragmentation and the severe morphological and ultrastructural changes observed in the parasite, ultimately leading to cell death.

This study demonstrated the antiparasitic activity of LQB-118 in all evolutionary stages of *T. cruzi*. However, we performed bioenergetic and ultrastructural tests in epimastigote forms of the parasite, which constitutes a limitation of the present work regarding the mechanism of action of the molecule. Although we can extrapolate some parameters to other evolutionary stages, studies in amastigotes and trypomastigotes should be performed in the future to better understand the mode of action of LQB-118 in *T. cruzi*.

## Conclusions

In conclusion, the data presented here demonstrate the anti-*T. cruzi* activity of the synthetic pterocarpanquinone LQB-118 *in vitro* in all evolutionary forms of the parasite, intracellular amastigote, trypomastigote and epimastigote. This antiparasitic activity appears to involve mainly interference with mitochondrial function and sheds light on the development of novel mitochondrial-targeted chemotherapeutic agents against Chagas disease. Further studies are needed to evaluate the therapeutic activity of LQB-118 in an experimental model of *T. cruzi* infection.

## Materials and Methods

### Pterocarpanquinone LQB-118 and Benznidazole

Pterocarpanquinone LQB-118 (Figure 1A, inset) was synthesized as previously described^24^ in the Laboratory of Bioorganic Chemistry at the Instituto de Pesquisas de Produtos Naturais, Universidade Federal do Rio de Janeiro, Brazil. Benznidazole, the reference drug for Chagas disease, was kindly provided by the Pharmaceutical Laboratory of the State of Pernambuco (LAFEPE, Pernambuco, Brazil). LQB-118 and benznidazole were dissolved in DMSO (Sigma-Aldrich) for the assays, and the final DMSO concentration did not exceed 0.2%.

### Parasites

Epimastigotes of the Dm28c strain (DTU I) were cultured with weekly passages in plastic flasks (25 cm^2^) at 27 °C containing 5 mL of BHI (brain-heart infusion) (Bacto, Becton, Dickinson and Company) medium supplemented with 30 μM hemin (Sigma-Aldrich) and 10% fetal calf serum (FCS).

### Isolation and Culture of Murine Peritoneal Macrophage

Macrophages were obtained from male Swiss mice (5–7 weeks old) by peritoneal lavage with 5 mL of cold RPMI (Cultilab) medium. Cells (2 × 10^6^/mL) were plated in 24-well plates (0.5 mL/well) containing glass coverslips or in 96-well plates (0.1 mL/well) and incubated at 37 °C with 5% CO_2_ for 24 h. Following this period, nonadherent cells were removed, and the monolayers were used for toxicity or infection assays. Animal experimental procedures were approved by the Ethics Committee on Animal Use of the Instituto de Biologia Roberto Alcantara Gomes (IBRAG) of the Universidade do Estado do Rio de Janeiro–UERJ (protocol 026/2022).

### *In Vitro* *T. cruzi* Metacyclogenesis

To induce *in vitro* differentiation of epimastigotes into metacyclic trypomastigotes, 7-day-old epimastigotes were harvested by centrifugation at 1500*g* and incubated in Triatomine Artificial Urine (TAU) medium (190 mM NaCl, 17 mM KCl, 2 mM MgCl_2_, 2 mM CaCl_2_, 4.2 mM NaHCO_3_, 0.2 M phosphate buffer, pH 6.0) at 5 × 10^8^ cells/mL for 2 h at 27 °C. Then, epimastigotes (5 × 10^6^ cells/mL) were incubated in TAU3AAG medium (TAU supplemented with 10 mM l-proline, 50 mM L-sodium glutamate, 2 mM L-sodium aspartate, and 10 mM d-glucose) for 4 days at 27 °C.^25^ Approximately 70–85% metacyclic trypomastigotes were obtained with the procedure.

### Mammalian Cell Toxicity and Intracellular Amastigote Activity

Macrophage toxicity was assayed in monolayers plated in 96-well plates and incubated with LQB-118 (0–80 μM) diluted in RPMI medium plus 10% FCS, in triplicate, for 72 h at 37 °C with 5% CO_2_. Controls consisted of macrophage monolayers incubated with RPMI medium plus 0.2% DMSO (corresponding to the vehicle concentration used in the highest dose of LQB-118) supplemented with 10% FCS, or incubated with the reference drug benznidazole (0–400 μM). After 72 h, cell viability was assessed by measuring the mitochondrial-dependent reduction of MTT [3-(4,5-dimethyl-2-thiazol)-2,5-diphenyl-2H-tetrazolium bromide] (Sigma-Aldrich) to formazan. Briefly, MTT (22 μL/well, 5 mg/mL) was added and incubated for 3 h at 37 °C with 5% CO_2_. Then, the plate was centrifuged, the medium was removed, and the formazan crystals were dissolved in 175 μL of DMSO. Absorbance was measured at 570 nm using a microplate spectrophotometer (μQuant, BioTek Instruments, Inc.). The half-maximal cytotoxic concentration (CC_50_) was determined by logarithmic regression analysis using GraphPad Prism 8 software.

For the antiamastigote assay, macrophage monolayers were infected with metacyclic trypomastigotes (5 parasites:1 macrophage ratio) for 24 h. Noninternalized parasites were removed by washing with warm PBS, and the infected macrophage monolayers were treated with LQB-118 (0–30 μM) diluted in RPMI medium plus 10% FCS for 72 h at 37 °C with 5% CO_2_. Controls consisted of infected macrophage monolayers incubated with RPMI medium plus 0.2% DMSO supplemented with 10% FCS, or with benznidazole (0–100 μM). The monolayers were then stained using the Quick Panótico method (Laborclin, Brazil), and at least 100 macrophages per sample were counted under light microscopy. Results were expressed as an (Infection Index = % infected macrophages × number of amastigotes/total number of macrophages). The half-maximal inhibitory concentration (IC_50_) was determined using the infection index and calculated by nonlinear logarithmic regression analysis with GraphPad Prism 8 software. Assays were performed in triplicate and repeated in three independent experiments.

### Evaluation of LQB-118 Activity against Epimastigotes and Trypomastigotes

Epimastigotes of *T. cruzi* were cultured in a 24-well culture plate (1 × 10^5^ cells/well) with LQB-118 (0–20 μM) in BHI medium supplemented with 30 μM hemin and 10% FCS at 27 °C for 96 h. Controls consisted of parasites incubated with BHI medium plus 0.2% DMSO supplemented with 10% FCS. The reference drug benznidazole (0–25 μM) was used as a positive control. The number of epimastigotes was counted daily in a Neubauer chamber. The half-maximal inhibitory concentration (IC_50_) was determined by nonlinear logarithmic regression analysis using GraphPad Prism 8 software.

Metacyclic trypomastigotes, obtained by in vitro metacyclogenesis, were incubated in RPMI medium plus 10% FCS in a 24-well plate (1 × 10^7^ cells/mL) at 27 °C for 48 h in the presence of LQB-118 (0–30 μM). Controls consisted of parasites incubated with RPMI medium plus 0.2% DMSO supplemented with 10% FCS or with the reference drug benznidazole (0–50 μM). Motile trypomastigotes were counted in a Neubauer chamber after 48 h. The half-maximal inhibitory concentration (IC_50_) was determined by nonlinear logarithmic regression analysis using GraphPad Prism 8 software.

### *In Situ* Detection of DNA Fragmentation in Intracellular Amastigotes

To evaluate whether treatment with LQB-118 induces DNA fragmentation in intracellular amastigotes, the analysis was performed using an in situ cell death detection kit (POD, Roche) according to the manufacturer’s instructions. Monolayers of peritoneal macrophages were prepared as previously described on Lab-Tek eight-chamber slides (Nunc, Roskilde, Denmark) and infected with metacyclic trypomastigotes (5 parasites:1 macrophage ratio) for 24 h. Noninternalized parasites were removed, and the infected peritoneal macrophages were treated with LQB-118 for 72 h at 37 °C with 5% CO_2_. Controls consisted of infected macrophage monolayers incubated with RPMI medium supplemented with 10% FCS plus vehicle (0.2% DMSO) or with 3000 U/mL DNase (Roche). Briefly, for in situ labeling of DNA fragmentation, the monolayers were fixed in 4% paraformaldehyde, incubated in a solution of 3% hydrogen peroxide in methanol, permeabilized with a solution of 0.1% Triton X-100 plus 0.1% sodium citrate, and then labeled with the TUNEL solution at 37 °C in the dark. Monolayers were counterstained with 300 μM 4′,6-diamidino-2-phenylindole (DAPI). Finally, the monolayers were washed with PBS and analyzed by fluorescence microscopy (Nikon Eclipse 80i).

### Effect of LQB-118 on the Infectivity of Metacyclic Trypomastigotes

Metacyclic trypomastigotes (1 × 10^7^ cells/mL) were previously treated with LQB-118 (10 and 40 μM) at 27 °C for 48 h. Then, monolayers of macrophages (obtained as previously described) were infected with pretreated trypomastigote (5 parasites:1 macrophage ratio) for 24 h at 37 °C/5% CO_2_. Following infection, one part of the coverslips of each sample (24 h of infection) was collected and stained with Quick Panotico. The other part of the infected macrophage monolayers was washed with PBS and incubated with RPMI supplemented with 10% FCS for another 48 h (totaling 72h of infection) and then stained. Results are shown as the Infection Index and images of infected macrophage monolayers were captured by light microscopy.

### Epimastigotes O_2_ Consumption after LQB-118 Treatment

Epimastigotes incubated for 5 days were transferred to fresh culture medium containing LQB-118 (2.5 or 25 μM) and further incubated for 24 or 48 h at 27 °C. Controls consisted of parasites incubated with BHI medium plus vehicle (0.2% DMSO) supplemented with 10% FCS. Then, parasites (2.5 × 10^7^ cells/mL) were washed with PBS and resuspended in BHI medium. Oxygen consumption was evaluated by high-resolution respirometry (Oxygraph-2K; OROBOROS Instruments, Innsbruck, Austria) at a constant temperature of 28 °C throughout the experiment. Oxygen concentration and flux were recorded using DatLab 7.4 software (Oxygraph-2K; OROBOROS Instruments, Innsbruck, Austria). Leak respiration was stimulated by adding 2 μg/mL of the ATP synthase inhibitor oligomycin (Sigma-Aldrich).

To induce the uncoupled state of maximum respiration, up to 1 μM carbonyl cyanide *p*-(trifluoromethoxy)­phenylhydrazone (FCCP) (Sigma-Aldrich) was added, thereby allowing the electron transport system to reach its maximal capacity. Respiration was inhibited by adding 2 μg/mL of Antimycin A (Sigma-Aldrich), a complex III inhibitor, to determine residual oxygen consumption (ROX). Physiological respiration (Routine), the electron transport system maximal capacity (ETS), and Leak respiration were calculated by subtracting the ROX value from the initial basal oxygen consumption after the addition of FCCP and oligomycin, respectively. Mitochondrial oxidative phosphorylation (OXPHOS) was calculated by subtracting Leak respiration from Routine. Spare respiratory capacity (SRC) was calculated by subtracting Routine from ETS. The flux control ratio (FCR), which reflects the fraction of the electron transport system maximal capacity corresponding to oxidative phosphorylation and to Leak respiration, was calculated by dividing OXPHOS/ETS and LEAK/ETS, respectively.^26^

### Measurement of H_2_O_2_ Production on Parasites Treated with LQB-118

Intracellular levels of H_2_O_2_ in epimastigotes were measured using the fluorescent probe Amplex Red (ThermoFisher). After 7-day-old of incubation, epimastigotes were harvested, transferred to a new culture medium containing LQB-118 (2.5 and 25 μM) for 24 and 48 h at 27 °C. Controls were parasites incubated with BHI medium plus vehicle (DMSO 0.2%) supplemented with 10% FCS. FCCP was used to evaluate if the H_2_O_2_ produced was mitochondrial. After 24 and 48 h of treatment, parasites (1.5 × 10^7^ cells/mL) were centrifuged and washed with PBS. Some samples were incubated with 2 μM FCCP for 30 min. Parasites were resuspended with a Working solution (0.1 mM Amplex red +0.2 UL/mL HRP (horseradish peroxidase-Sigma)) and incubated for 30 min in the dark. Cells were centrifuged, and 100 μL of suspension, with the resorufin produced, was plated in triplicate in a 96-well dark plate. The fluorescence was measured using an EnvisionR 2104 Multilabel Reader (PerkinElmer) with an excitation wavelength of 570 nm and an emission wave of 585 nm. H_2_O_2_ concentrations were determined using a standard curve prepared by incubating increasing concentrations of H_2_O_2_ with the working solution for 30 min, followed by fluorescence measurement.

### Ultrastructural Analysis of Epimastigotes after Treatment with LQB-118

Epimastigotes (1 × 10^7^ cells/mL) were incubated with LQB-118 (2.5 and 25 μM) for 48 h at 27 °C. Controls consisted of parasites incubated with BHI medium plus vehicle (0.2% DMSO) supplemented with 10% FCS. For scanning electron microscopy, parasites were adhered to poly-l-lysine-coated coverslips for 30 min and fixed with Karnovsky’s solution (4% paraformaldehyde, 2.5% glutaraldehyde in 0.1 M Na-cacodylate buffer, pH 7.2) at room temperature for 24 h. Cells were washed twice in 0.1 M cacodylate buffer (pH 7.2), postfixed in 1% OsO_4_ plus 0.8% K_3_Fe­(CN)_6_, washed again in 0.1 M cacodylate buffer (pH 7.2), dehydrated in a graded ethanol series (20–100% v/v) for 30 min at each step, critical-point dried in CO_2_, mounted on metallic stubs, and coated with a 20 nm gold layer. Samples were examined using a JEOL JSM-7100F field emission scanning electron microscope. For transmission electron microscopy, after treatments, chemically fixed samples were washed in 0.1 M cacodylate buffer (pH 7.2), postfixed in 1% OsO_4_ and 0.8% K_3_Fe­(CN)_6_ for 40 min at room temperature, washed again in 0.1 M cacodylate buffer (pH 7.2), dehydrated in a graded ethanol series (20–100% v/v) for 20 min at each step, and embedded in Polybed 812 epoxy resin. Ultrathin sections (70 nm) were obtained using a Leica UC7 ultramicrotome, collected on copper grids, stained for 30 min in 5% aqueous uranyl acetate, and for 3 min in 1% lead citrate. Analyses were performed using a Hitachi HT 7800 transmission electron microscope.

### Statistical Analysis

Statistical analyses were conducted with GraphPad Prism 8 software (GraphPad Software, Inc., San Diego, CA). Data were presented as the mean ± standard error (SE), and all experiments were independently performed at least three times. Data were analyzed by one-way ANOVA, and differences among groups were assessed using Tukey’s post-tests. An unpaired Student’s *t* test or Mann–Whitney test was used when necessary.
